# The possible puzzles of BCG vaccine in protection against COVID-19 infection

**DOI:** 10.1186/s43168-021-00052-3

**Published:** 2021-01-27

**Authors:** Basheer Abdullah Marzoog, Tatyana Ivanovna Vlasova

**Affiliations:** 1 Orgdivision National Research Mordovia State University, Bolshevitskaya St., 68, Saransk, Rep. Mordovia 430005 Russia; 2 Department of Normal and Pathological Physiology, National Research Mordovia State University, Saransk, Russia

**Keywords:** COVID-19, SARS-CoV2, BCG, *Mycobacterium tuberculosis*

## Abstract

**Background:**

The paper aimed to analyze and evaluate the present literature data on the clinical effectiveness of using the bacillus Calmette–Guérin (BCG) vaccine in protecting against the novel coronavirus disease 2019 (COVID-19).

**Main body:**

Several novel clinical data have shown a relationship between the vaccinated population with the bacillus Calmette–Guérin (BCG) vaccine and the severity and mortality rate from coronavirus disease 2019 (COVID-19). However, the linkage between the BCG vaccine and COVID-19 infection mortality and morbidity rate is still ambiguous. The BCG has been protected previously from many other respiratory viral infections. The efficacy of the BCG vaccine in the protection against COVID-19 depends on various factors including social, economic, cultural norms, mitigation efforts, health infrastructure, and demographic differences between countries.

**Conclusion:**

Thus, the literature analyses show a noticed difference between the countries that follow national vaccination programs than in countries that do not follow such programs (Italy, Netherlands, USA). However, there are not any recommendations for using BCG in the protection against severe cases of COVID-19. The severity of COVID-19 maybe depends on the age, immune state of the patient, and the level of vaccine coverage. The possible reason for BCG protection is trained immunity in both diseases.

## Background

The novel COVID-19 pandemic is a global emergency that required a worldwide response to control the progression of the novel infection and try to limit its expansion. The COVID-19 is an acute lower respiratory system infection results after getting the severe acute respiratory syndrome coronavirus 2 (SARS-CoV2) virus. The first reported case of COVID-19 was on 30 December 2019 in Wuhan, China [1]. On the evening of October 28, 2020, reported 43,979,777 cases worldwide and 1,167,124 death related to coronavirus infection [2]. What is in common between the TB and COVID-19? It seems to be the cytokine storm and the improper response of the immune system against both infections are dominant [3]. Primarily, CD4+ T cells, interferon γ, and tumor necrosis factor are extremely important for our anti-COVID-19 immunity. Also, probably important are CD8+ T cells, γδ T cells, CD-1 restricted T cell, interleukin-17, and interleukin-2. Moreover, it may be relevant for our immunity against COVID-19 infection B cells and antibodies too. The BCG is protective against pulmonary tuberculosis in about 60% of cases [4]. The BCG is generally safe with the main side effect of the development of inflammation at the site of injection. The paper is dedicated to analyze and discuss briefly the present data on the association between the BCG vaccination and the COVID-19 epidemiology since it is an urgent situation.

## Main text

Bacillus Calmette–Guérin (BCG) vaccine is used to protect from *Mycobacterium tuberculosis* (Mtb) infection since 1921 in children, but unfortunately, it is not effective in adults [5]. Recently, new data shared by the *Nature* journal explained the reasons for the ineffectiveness of BCG vaccine in young and adults, due to the failure in the control of the infection and delayed response of the mucosal dendritic cells (DCs) in presenting antigen of the Mtb to the CD4+ T cell which allow the bacteria to divide (time factor), and not due to a weak response. Moreover, the external supply of activated *Mtb* antigen-primed DCs into vaccinated mice, at the time of *Mtb* exposure have successfully induced CD4+ T cell response and eliminated early *Mtb* growth [6]. The tuberculin proteins are used to diagnosis tuberculosis, and it is recommended to perform before administration of the BCG vaccine, to prevent complications due to pre-existing immunity to mycobacterial antigens [7]. Variable types of BCG vaccine are administered to the newborns: whole organism or subunit, choice of vector and antigen. The BCG effectiveness in protection against tuberculosis potentially variable depends on the strains of BCG, age at vaccination, nutrition, and exposure to environmental mycobacteria. Many countries have discontinued the use of universal vaccination policies due to the low risk of development of *Mycobacterium bovis* infection. BCG vaccine induces an immune response against *Mycobacterium tuberculosis* via CD4+ T helper cell and CD8+ T cytotoxic cell [8]. A small clinical study performed by applying the BCG vaccine for healthy volunteers before administration of the seasonal influenza vaccine has shown a pronounced increase and acceleration in the induction of functional antibody responses against the 2009 pandemic influenza A (H1N1) [9]. For many years, scientists believed that innate immunity cannot adapt and learn from previous infections until recently, and there was evidence on the capacity of the innate immunity to effectively and strongly respond to the previous infection and even efficiently activate the adaptive immunity [10]. This referred to some epigenetics and metabolic reprogramming of the immune cells including the natural killer, neutrophils macrophages, and other white blood cells [11–13]. The BCG has shown a capacity to induce the so-called trained immunity that can protect against the non-mycobacterial infections compromising: staphylococci, candidiasis, yellow fever, and influenza [14]. Therefore, the BCG holds a wide range of capabilities and more puzzles to be solved.

### The role of BCG vaccination in COVID-19 mortality rate: the possible mechanism

Undeniably, the BCG contributes to the protection and lowering of the severity of the novel COVID-19 infection through triggering innate immunity particularly via enhancing macrophage activity and CD4+ T cells. Several studies presented a correlation between BCG vaccination and COVID-19 mortality under different scenarios [15]. Using of variable types of BCG vaccine may play a role in determining the effect of BCG during the pandemic via inducing an innate immune response, inducing cytokine secretion in peripheral blood lymphocytes, particularly, robust proliferation of CD4+ and CD8+ T cells, higher secretion of Th1 cytokines (interferon-γ, TNF-α, and IL-2), and low secretion of Th2 cytokines (IL-4). Thus, a particular BCG strain might preferentially act on the immune system in a manner that significantly reduces the morbidity/mortality associated with certain viral infections. A small study concluded that the COVID-19 deaths per million are negatively associated with the percentage of BCG coverage indicating that every 10% increase in the BCG index was associated with a 10.4% reduction in COVID-19 mortality [15]. Furthermore, there was a moderate correlation between the time of universal BCG vaccination and population density, while the percentage of population > 65 years of age and the Human Development Index are strongly correlated. In Holland and Australia, there are ongoing randomized clinical trials to vaccinate the health care providers with BCG and placebo to show its efficacy in protection against COVID-19 [16, 17]. A single study done in Denmark hypothesized that BCG vaccination can reduce health care providers’ absenteeism during the COVID-19 epidemic through non-specific effects (NSEs) of BCG [18]. Therefore, the health care providers are now considered a candidate for receiving the BCG despite the availability of effective COVID-19 vaccines to prevent TB and achieve maybe a better response against COVID-19 in combination with the COVID-19 vaccine. The BCG vaccine could decrease the severity of COVID-19 infection through the trained immunity via the promotion of genetic regions encoding for pro-inflammatory cytokines, particularly interleukin-1B that plays a key regulatory role in reducing respiratory infections and sepsis in addition to its antiviral immunity [19–23]. Therefore, the BCG protection activity could not be due to its direct action against COVID-19 but through reducing sepsis and infections co-occurring [5]. An extremely important role played by the BCG in the immunotherapy, while its direct administration into the tumors is used to treat superficial bladder carcinoma via inflammation triggering approach [24]. Besides, a shred of evidence of a BCG-based efficacy in patients allergic asthma, multiple sclerosis, alopecia areata, oral and cutaneous lichen planus, and type I diabetes mellitus, since the vaccine has been linked to reduced blood sugars (through accelerating glucose utilization), changes in metabolism, and epigenetic changes in T regulatory genes [25]. This endorses the hypothesis that the BCG effect is not limited to a specific disease but it modifies the immune system response.

## Conclusion

To date, most of the published studies show an association between the BCG vaccination and the severity of COVID-19 infection [26–32]. The relation of universal BCG vaccination programs and COVID-19 severity could be due to single exposure to an attenuated pathogen during infancy result in a lifelong enhancement in immune surveillance. These findings are the basis for the initiation of BCG vaccination trials to fight infections such as COVID-19. However, the complete linkage is still unclear and no clinical trials recommend the use of the BCG vaccine in COVID-19. Therefore, it is necessary to perform more clinical researches on the BCG attribution to the COVID-19 to reveal the remaining portion of the plot.

After the presence of effective vaccinations against COVID-19, now the studies turn to whether the BCG vaccine enhances the induction of immune response against COVID-19, if combined with the novel COVID-19 vaccines or not? Hypothetically, it is not surprising that BCG will induce the effect of vaccine efficacy through its non-specific effects. But for proofing, it required extra clinical data and research.

Now it is understood that the BCG vaccine exerts a wide range of effects not only against TB but also on other non-TB-related pathologies through its capacity to modify cells of the innate immune system to create a memory for the pathogen without antibodies.

## Data Availability

Not applicable

## Abbreviations

COVID-19: Coronavirus disease 2019
BCG: Bacillus Calmette–Guérin
Mtb: *Mycobacterium tuberculosis*
SARS-CoV-2: Severe acute respiratory syndrome coronavirus 2
